# Background Light Rejection in SPAD-Based LiDAR Sensors by Adaptive Photon Coincidence Detection

**DOI:** 10.3390/s18124338

**Published:** 2018-12-08

**Authors:** Maik Beer, Jan F. Haase, Jennifer Ruskowski, Rainer Kokozinski

**Affiliations:** 1 Fraunhofer Institute for Microelectronic Circuits and Systems, 47057 Duisburg, Germany; jan.haase@ims.fraunhofer.de (J.F.H); jennifer.ruskowski@ims.fraunhofer.de (J.R.); 2 Department of Electronic Components and Circuits, University Duisburg-Essen, 47057 Duisburg, Germany; rainer.kokozinski@uni-due.de

**Keywords:** light detection and ranging (LiDAR), time-of-flight (TOF), single-photon avalanche diode (SPAD), CMOS, system-on-chip (SoC), background light rejection

## Abstract

Light detection and ranging (LiDAR) systems based on silicon single-photon avalanche diodes (SPAD) offer several advantages, like the fabrication of system-on-chips with a co-integrated detector and dedicated electronics, as well as low cost and high durability due to well-established CMOS technology. On the other hand, silicon-based detectors suffer from high background light in outdoor applications, like advanced driver assistance systems or autonomous driving, due to the limited wavelength range in the infrared spectrum. In this paper we present a novel method based on the adaptive adjustment of photon coincidence detection to suppress the background light and simultaneously improve the dynamic range. A major disadvantage of fixed parameter coincidence detection is the increased dynamic range of the resulting event rate, allowing good measurement performance only at a specific target reflectance. To overcome this limitation we have implemented adaptive photon coincidence detection. In this technique the parameters of the photon coincidence detection are adjusted to the actual measured background light intensity, giving a reduction of the event rate dynamic range and allowing the perception of high dynamic scenes. We present a 192 × 2 pixel CMOS SPAD-based LiDAR sensor utilizing this technique and accompanying outdoor measurements showing the capability of it. In this sensor adaptive photon coincidence detection improves the dynamic range of the measureable target reflectance by over 40 dB.

## 1. Introduction

To bring autonomous vehicles to the road, a fast and reliable high-resolution perception of the environment is essential. Today, cars include many different driver assistance systems, like adaptive cruise control, lane assist, or emergency braking. However, all these systems are designed to assist the driver and make the journey more comfortable. In case of a system malfunction, it is disabled and the driver has to undertake the task. In self-driving cars there is no human driver to replace non-functional systems and, therefore, much higher reliability requirements are demanded. Different sensors are used for environmental perception [1]: ultrasound for short-range applications, like parking assistance [2,3], 2D cameras for lane assists, and stereo vision and radar for long-range applications, like collision warning and emergency braking systems [4,5]. Since radar suffers from low angular and low distance resolution, the most emerging sensor technology for self-driving cars is light detection and ranging (LiDAR) [6,7]. LiDAR is a technique for high angular and high depth resolution 3D imaging. LiDAR systems are based on measuring the time-of-flight (TOF) of an emitted and reflected laser signal in the visible or near infrared spectrum [8]. Due to the short wavelength a high angular resolution similar to 2D image sensors can be achieved. High resolution gives several opportunities to improve the environmental perception by image processing, like the detection and location of small obstacles, the classification and tracking of objects, or the estimation of their velocity and moving direction. Especially in safety-critical applications these possibilities can help to increase the reliability of automated systems. Therefore, LiDAR is seen as the most promising technology for autonomous driving [9]. Nevertheless, automotive applications impose high requirements on the systems: they need to be cost-efficient, durable, and operational in all environmental conditions. To reduce cost and increase durability, solid-state sensors can be fabricated in well-established standard CMOS technology. Since silicon-based detectors are only sensitive up to a wavelength of around 1100 nm [10], laser sources between 850 nm and 950 nm are common. Therefore, ambient light coming from the sun is a major impediment for LiDAR in outdoor applications.

Different methods for the rejection of high ambient light are used in LiDAR systems. A basic approach is the use of optical bandpass filters adapted to the wavelength of the illumination source [6,11]. These filters remove most of the background light. Since commercial laser sources exhibit a certain emission bandwidth, fabrication induced variation, and temperature dependence, the filter bandwidth has to be chosen carefully to not sacrifice available laser power [12]. Another common approach to reduce the influence of high ambient light is the accumulation of multiple time measurements. If several single timestamps are collected in a histogram, the events pile up at the arrival time of the reflected laser pulse allowing a more reliable distance determination [13]. Obviously, increasing the number of accumulated timestamps improves the quality of the measurement, but lowers the frame rate at the same time. Therefore, a trade-off between frame rate and reliability has to be made. Another approach to cope with strong background light is the use of scanning lasers which illuminate only a single spot or line of the target scene at once [14]. In this technique the field-of-view of the laser source is much smaller compared to flash illumination allowing a higher optical power density and, hence, an easier distinction between laser signal and background light. Nevertheless, LiDAR systems based on scanning lasers are more expensive and less durable due to the need for beam steering, which can be realized by mechanical mirrors, micro electromechanical systems [15], or phase arrays [16].

In this paper we present a technique to improve the measurement performance at high ambient light. This technique can be implemented in addition to the aforementioned techniques for ambient light rejection and is especially suitable for single-photon avalanche diode (SPAD)-based LiDAR sensors. By detecting temporal correlated single photons in each pixel, false detections caused by ambient photons are reduced and, at the same time, an improvement of the signal quality is achieved allowing for higher measureable distances. Since fixed coincidence parameters as usually used in the literature [11,17,18] show good measurement results only within a small range of target reflectance and ambient light intensity, respectively, we use adaptive coincidence detection. In this technique the parameters of the photon coincidence detection are adjusted to the actual ambient light intensity. This allows for covering a much higher dynamic range in target reflectance. Applying the parameter adjustment pixel-individual enables the LiDAR system to capture whole daylight scenes in a single shot. In this paper we explain the basic limitation induced by high ambient light and show how photon coincidence detection can improve the measurement. We present a 192 × 2 pixel dual-line SPAD-based LiDAR sensor fabricated in an automotive-certified 0.35 µm CMOS process with multiple SPADs in each pixel to detect photon coincidences. For validation of the proposed adaptive coincidence detection technique a flash LiDAR camera has been developed. The implementation of the coincidence controlling algorithm along with outdoor measurement results will be presented. The measurements prove the feasibility of improving the measureable dynamic range of target reflectance by adjusting the coincidence parameters to the ambient light conditions.

## 2. Background Light in SPAD-Based LiDAR

**Figure 1:**
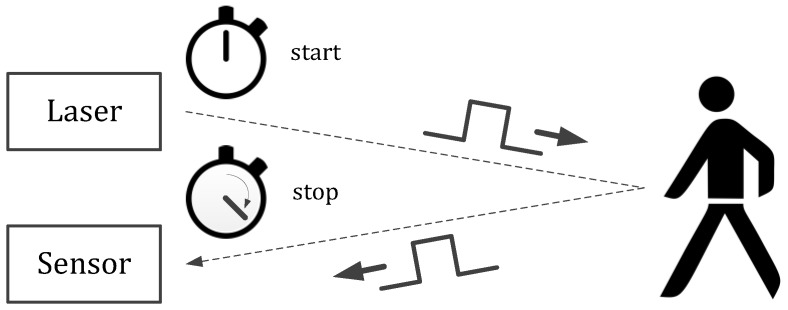
Operation principle of the direct time-of-flight measurement technique. By a high precision electronical stopwatch the time between emission and reception of a short laser pulse is captured.

In the direct TOF measurement technique the time between emission and reception of a laser pulse is measured by a high precision electronical stopwatch as shown in Figure 1 [8]. On-chip time measurement can be performed by time-to-digital (TDC) or time-to-analog converters. Usually the time measurement starts with the emission of the laser pulse and stops at the first detected photon. Since the received optical power of the reflected laser signal scales inversely quadratic with the distance, highly sensitive photodetectors are required for long range and low emission power applications [19]. SPADs use avalanche multiplication, which is a prompt process, to obtain a large signal response to the faint reflected light. This allows the detection of single photons with a time resolution in the picosecond range [10]. Therefore, SPADs are eminently suited for long-range automotive LiDAR applications. Since the statistical fluctuation of the first detected photon arrival time increases with lower detector sensitivity, higher photon detection efficiency (PDE), defined as the probability to detect an incident photon, reduces the variance and improves the measurement accuracy. The PDE takes into account the photon absorption, the avalanche triggering probability and, in the case of pixel arrays, the fill factor of the sensor [20]. Since no ideal photodetector is available, an additional uncertainty is added to the TOF measurement. To deal with this problem usually several time measurements are accumulated in a histogram [16] to increase the probability to capture the first arriving photon and obtain an accurate measurement. To summarize, if no background light or dark count rate is taken into account, a higher PDE improves the measurement accuracy and range. Using shorter laser pulses with higher peak power improves the precision as well, since this also increases the probability to capture a photon close to the true TOF. However, since achieving narrow laser pulses becomes more difficult for increasing peak power, there is a certain technical limitation. Furthermore, for many applications eye safety regulations have to be fulfilled limiting the laser pulse energy and repetition rate [16].

Unfortunately, background illumination and dark counts are always present in real LiDAR systems; especially in outdoor applications high background light is present. Since background photons impinge the sensor during the whole measurement cycle starting right at the laser pulse emission, there is a certain probability for background photons to arrive at the detector before the reflected laser pulse and cause false detections [21]. The probability of such false detections depends on the distance of the object to be measured, as well as on the background light photon detection rate. To show the effect of high ambient light, we take a look on the detection probability of the first photon. With the time dependent photon detection rate R(t) the probability density function (PDF) of the first event can be calculated according to [21]:

(1) $$P_{1}\left(t\right)=R\left(t\right)\left(1-\overset{t}{\underset{0}{\int }}P_{1}\left(\tau \right)d\tau \right).$$

**Figure 2:**
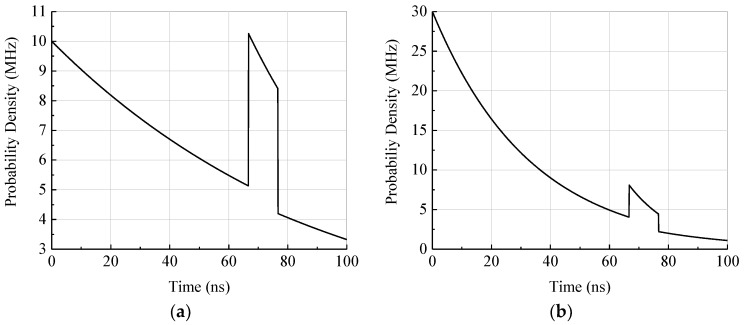
(**a**) Probability density of the first photon detection for measurement duration of 100 ns, a pulse width of 10 ns, and a TOF of 67 ns. The photon rates are assumed to be 10 MHz for the laser pulse and background light; and (**b**) the probability density for photon detection rates of 30 MHz achieved by increasing the PDE or target reflectance by a factor of three.

In laser light, as well as sunlight, the photon inter-arrival times are exponentially distributed [22]. This can be obtained from (1) by assuming a constant photon rate. For the following examples we assume constant ambient and laser light intensities. The calculated PDF depends on the photon detection rates of the sensor only. The mentioned photon detection rates are defined as the rate of actually-detected photons. Assuming appropriate circuitry, the SPAD generates a digital pulse for each detected photon and, therefore, the rate corresponds also to the signal pulse rate. The rate takes into account the applied laser source, the geometrical metrics of the sensor, the used optics or filters, the target conditions, and SPAD characteristics. In Figure 2 the calculated probability density according to Equation (1) for the first photon detection as a function of time is shown. In the example a total measurement range of 100 ns, a pulse width of $T_{P}=10\;\mathrm{ns}$, and a TOF of $T_{\mathrm{TOF}}=67\;\mathrm{ns}$, which corresponds to an object distance of 10 m, are assumed. For Figure 2a photon detection rates of $R_{B}=R_{L}=10\;\mathrm{MHz}$ are used for the laser pulse and ambient light. In this case the laser pulse can be clearly separated from the background noise. Figure 2b shows the same situation with photon rates of $R_{B}=R_{L}=30\;\mathrm{MHz}$ corresponding to a three times higher PDE or target reflectance. In this case the laser pulse is much smaller in comparison to the ambient noise and, therefore, more difficult to locate. Since the photon counts in each bin of the histogram are binomial-distributed, which can be approximated by the Poisson distribution for low bin-wise detection probabilities, the expected values and probability, respectively, directly correspond to the variance of the bin count. For this reason the laser pulse in Figure 2b is more difficult to locate even if the step size in probability density at the pulse time-of-arrival is comparable to Figure 2a.

The probability for the time measurement to be stopped before the reflected laser pulse returns, which means the system is unable to capture the true TOF, is given by the integral of the PDF from zero to the TOF. Since this probability increases with the object distance and ambient photon detection rate, higher ambient photon rates are tolerable at shorter distances, and vice versa [21]. In the examples the probabilities of false detections are calculated as 48.8% and 86.6%, respectively. Based on the PDF according to Equation (1) we have defined a signal-to-noise ratio (SNR) of the first-photon direct TOF measurement. It is given by the number of detected signal photons over the standard deviation of all photons detected during the pulse arrival time. Plotted versus the target reflectance (i.e., constant distance), the SNR has a clear maximum at:

(2) $$R_{B}=1/T_{\mathrm{TOF}}$$

where $T_{\mathrm{TOF}}$ is the TOF of the emitted laser pulse [23]. Depending on the application, as well as the optics of the LiDAR system, an improvement of the SPAD PDE is not required as long as this optimum background photon detection rate is obtained. Obviously, to obtain the optimum photon detection rate the sensor’s sensitivity can be adjusted in other ways, such as by using different apertures or gray filters. Nevertheless, a higher PDE can enable improvements on the system: specific detection and ambient light rejection methods may require a higher photon detection rate and, therefore, need a higher detector PDE. In first-photon direct TOF measurement systems a trade-off in sensitivity is required: a higher PDE improves the range and precision, but increases the amount of false detections caused by high background light.

In conclusion, for applications at high ambient illumination the ambient photon detection rate must not exceed a certain level. Since the best measurement results are achieved at a certain distance dependent ambient photon detection rate according to Equation (2), a higher PDE, which increases the ambient photon rate, could result in a loss of measurement performance if the ambient photon rate is thereby increased above the optimal level. To counteract the high ambient photon rate due to high PDE or high ambient illumination, the rate has to be kept on the optimal level by rate adjustment. In our sensor this is achieved by photon coincidence detection whereas the parameters of the coincidence detection are adjusted to the actual ambient photon detection rate.

## 3. Photon Coincidence

**Figure 3:**
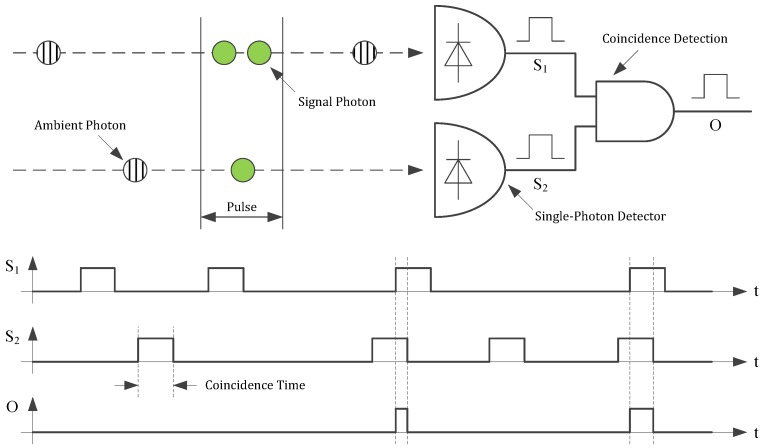
Simple circuit for the detection of photon coincidences. If the output pulses of the detectors with a width corresponding to the coincidence time overlap, a coincidence event is detected.

To reduce the amount of false photon detections in real-time without sacrificing range and precision, photon coincidence detection can be applied. In this technique the measurement is not stopped by the detection of a single photon, but only if at least a defined number of single photons—called coincidence depth—is detected within a defined timespan—called coincidence time. Since the laser photons are confined to the pulse width whereas the background photons are approximately equally distributed in time, the ability to differentiate between ambient and laser signal is improved [24,25]. Compared to the case of narrowing the aperture or using a gray filter, this technique improves the range of the systems in high ambient light applications. In Figure 3 a simple circuit for coincidence detection with a coincidence depth of two is shown. Each time a photon is detected, a pulse corresponding to the coincidence time is generated at the detector output. To find coincidences the output signals of the detectors are connected by an AND gate. Thus, if the output pulses overlap, which happens only if the pulses are separated by less than the coincidence time, the output of the AND gate is set high indicating the detection of a coincidence event as shown in the timing diagram [26]. This circuit principle can be expanded to higher coincidence depths.

The basic idea of this principle is to apply a certain threshold and stop the time measurement as soon as the impinging light intensity and photon rate, respectively, exceeds this threshold. In a best-case scenario this level is set slightly above the ambient light intensity. Since the laser pulse adds to the ambient light, the received light intensity increases, exceeds the threshold, and stops the time measurement. Unfortunately, the photons do not arrive in regular intervals but the inter-arrival time obeys the exponential distribution. Therefore, a total rejection of ambient light is not possible. However, since low photon rates (i.e., only ambient light) are more reduced than higher photon rates (i.e., laser pulse with ambient light) this technique increases the ratio of the laser signal and ambient light event rate (signal-to-background-ratio, SBR) allowing a longer distance range at high ambient light [23]. An event is an incident used to stop the time measurement. Depending on the applied detection mechanism, an event can be the detection of a single photon or a photon coincidence comprising several single photons.

**Figure 4:**
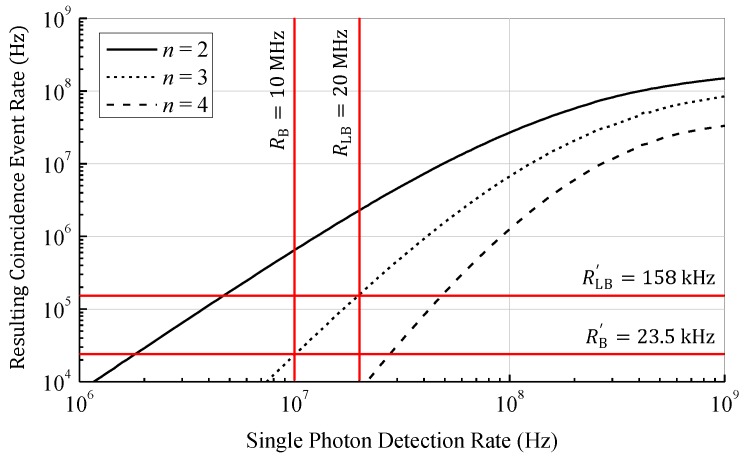
Photon coincidence event rate as a function of the single photon detections according to a statistical coincidence model based on photon inter-arrival times calculated for a coincidence time of 10 ns. The model takes into account the coincidence time, the coincidence depth, the number of SPADs, and the dead time of the SPADs. Highlighted values illustrate the improvement in SBR.

To investigate the effect of photon coincidence detection on the event detection rate, a model based on statistical calculations has been developed. This model is an advanced version of the model presented in [23] and additionally takes into account the dead time of the SPADs. The dead time is relevant since the SPADs are quenched and reset after each single photon detection. Since the SPADs are insensitive to further incident photons during this phase, the dead time limits the maximum single photon detection rate. To allow coincidence times below the dead time of the SPAD, multiple SPADs are combined for the detection of photon coincidences. Therefore, the model is determined from the PDF of the individual photons detected in an array of dead time afflicted SPADs. In Figure 4 the rate of photon coincidence detections as a function of the ideal single photon detection rate (i.e., without saturation effects, like dead time) of the whole array according to our model is shown for an array containing four SPADs, a coincidence time of 10 ns, and a coincidence depth n between 2 and 4. As expected, with increasing coincidence depth the rate of coincidence events decreases due to the lower probability of receiving at least n photons within the coincidence time. For rising coincidence depth a steeper slope of the curves is observable corresponding to a gain in SBR. To fully eliminate the ambient light, the slope should be infinity and located between the photon rates of ambient light and ambient with laser light. Even if an infinite slope is not possible, the steepness of the slope can be increased by increasing the coincidence depth n as shown in Figure 4. However, higher coincidence depths require higher single photon rates. Due to the dead time of the SPADs the maximum single photon detection rate is limited and the curves saturate. Therefore, to increase the range by increasing the coincidence depth a short dead time is required. On the other hand, a short dead time increases the afterpulsing probability which counters the benefit of photon coincidence detection.

As an example arbitrary input values are chosen and highlighted in Figure 4. With a photon rate of the ambient light and laser pulse of 10 MHz each, we get photon rates of $R_{B}=10\;\mathrm{MHz}$ for the ambient light and $R_{L}+R_{B}=R_{\mathrm{LB}}=20\;\mathrm{MHz}$ during the laser pulse (i.e., ambient and laser photons). In this case the SBR given by $R_{L}/R_{B}$ is 1. If photon coincidence with a coincidence time of 10 ns and a coincidence depth of n=3 is applied, the ambient event rate reduces to $R_{B}^{\prime }=23.5\;\mathrm{kHz}$ and the event rate during the laser pulse to $R_{\mathrm{LB}}^{\prime }=158\;\mathrm{kHz}$. The SBR can now be calculated as 5.72. Since the SBR directly affects the range of the LiDAR system, a higher SBR corresponds to a longer distance range. In case of zero background light the initial SBR without photon coincidence is infinity and, therefore, photon coincidence does not improve the range in low ambient light applications. Since the dark count rate of modern CMOS SPADs is below 10 cps/µm^2^ [27], it is neglectable compared to the background light in typical outdoor environments.

As can be recognized in Figure 2, even if the ambient light intensity is constant, the maximum range is only achieved if the event detection rates are chosen deliberately by adjusting the light reception mechanism. If the event rates are too high, mostly ambient events are detected. On the other hand, if the rates are too low, almost no events are generated by the reflected laser signal. In both cases the laser signal cannot be located in the histogram and the distance measurement fails. Since the event rates change due to different target reflectance and ambient light conditions, a fixed ratio between light intensity and event rate works well for a specific target object only. To cover a wide range of different targets the coincidence parameters in the presented sensor are designed to be variable. By adjusting these parameters based on the present background light and target conditions, the resulting event rate of the background light $R_{B}^{\prime }$ could be kept constant. According to Figure 4 a resulting event rate of 1 MHz can be achieved up to a single photon rate of around 100 MHz. This gives an improvement in dynamic range of 40 dB. By adjusting other coincidence parameters like coincidence time as well, the dynamic range can be further improved. Adaptive photon coincidence parameter adjustment allows a much better measurement performance at varying target conditions. Since the parameters can be adjusted in each pixel individually, scenes with high dynamic range can be captured in a single shot.

## 4. CMOS Flash LiDAR Sensor and Camera

**Figure 5:**
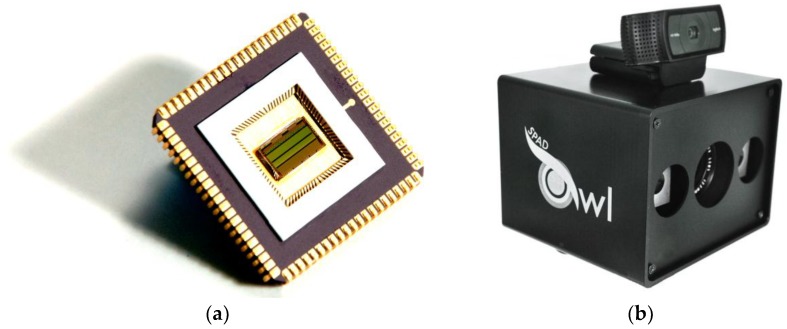
(**a**) Photograph of the CMOS SPAD-based flash LiDAR sensor with 192 × 2 pixel in a CQFJ84 package; (**b**) Flash LiDAR camera demonstrator “Owl” developed at Fraunhofer IMS.

With CMOS integrated SPADs the photodetector can be integrated along with the control and processing electronics on a single chip. This allows the realization of application specific sensors with dedicated CMOS circuitry. Additionally, system-on-chip sensors are more durable and can be fabricated more cost-efficiently than sensors built in other technologies or compound sensors. In this technology a LiDAR sensor [28] based on CMOS SPADs [10] has been developed and fabricated. The sensor in Figure 5a has two lines with 192 pixels each, integrated in-pixel circuitry for active SPAD quenching and reset, adjustable photon coincidence detection circuitry, in-pixel flash TDC with a temporal resolution of 312.5 ps, and readout electronics.

**Figure 6:**
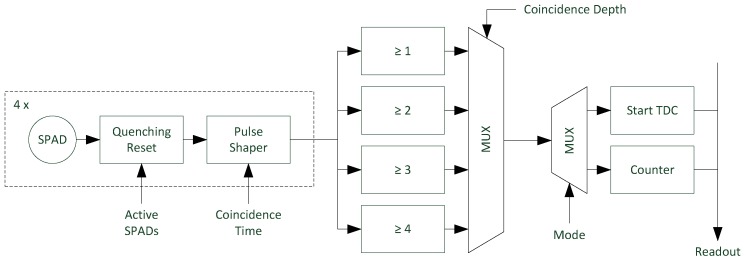
Block diagram of the sensor pixel. Each pixel uses four SPADs for the detection of photon coincidences. The coincidence time is adjusted by a variable pulse shaper and the coincidence depth by choosing one of the four different logical circuits processing the four SPAD outputs.

As shown in the pixel block diagram in Figure 6, each pixel of the sensor contains a digital silicon photomultiplier (dSiPM) with four vertically-arranged SPADs with a diameter of 12 µm and a fill factor of 5.32%. The SPADs exhibiting a PDE of 2% at 905 nm, a dark count rate of 10 Hz, and a dead time of 20 ns are connected by logical circuits for the detection of photon coincidences. To allow coincidence depth adjustment logical circuits for different depths connected to a multiplexer are used. The operational principle of these circuits is analogously to the circuit shown in Figure 3, but for different depths and with four inputs each. In timing mode the output of the multiplexer is connected to the TDC and triggers it as soon as the required number of single photons is detected. The coincidence time is defined by the width of the input pulses of the logic circuits which can be varied by an integrated pulse shaper with variable width. The pulse width is identical for all four SPADs and can be set to four values between 1.5 ns and 16 ns in our sensor. An additional parameter to adjust the resulting event rate is the number of SPADs used for the coincidence detection, which can be adjusted by disabling the reset of the SPAD. Since the number of used SPADs has to be at least equal to the set coincidence depth and without applied coincidence (depth = 1) the coincidence time has no effect, overall 28 different coincidence parameter sets are available.

For measurements and demonstration of the sensor we have built the flash LiDAR camera “Owl”. The camera shown in Figure 5b integrates the sensor with corresponding lens, an FPGA for sensor control and data readout, and two pulsed laser sources. The beam shaping optics of the laser sources are designed to match the field-of-view (FOV) of the two sensor lines. The lasers emit at 905 nm wavelength with 75 W peak power, 10 kHz pulse repetition rate, and 15 ns pulse width resulting in a mean optical emission power of 11.25 mW. The camera uses a 12 mm lens resulting in a FOV of 36° × 1° for each line of the sensor. With 192 pixels we end up with a pixel FOV of around 0.2° × 1°. In the typical operation we accumulate 400 single timestamps for distance determination resulting in a frame rate of 25 fps. For visualization of the measurement results, a webcam is mounted on top for 2D image acquisition allowing a superposition of the 3D and 2D data. Since the sensor features an event counting mode, it is able to generate 2D images as well, but two single lines are less descriptive. For future area sensors the webcam will be dispensable.

In addition to 2D image acquisition, the event counting mode is used to gain information about the ambient light intensity and event rate, respectively. In counting mode an integrated eight-bit counter is used allowing counting up to 255 events in a single measurement. In counting mode the output of the coincidence detection circuit, which triggers the TDC in the time measurement, is directly fed to the counter as shown in Figure 6. In this way the event rate is measured using the same coincidence settings as applied in the time measurement. Since the width of the counting window is known, the number of counted events directly corresponds to the event rate. To prevent a reduction of the frame rate, background light event rate measurements are performed between two consecutive laser pulses. The time window for event counting has to be chosen carefully to avoid counter overflow. Since the number of counted events is Poisson distributed, multiple measurements can be executed between each laser pulse to reduce the variance of the rate measurement. The measured event rates act as input signal for the adaptive photon coincidence processing. The algorithm adjusts the photon coincidence parameters to keep the resulting ambient light event rate $R_{B}^{\prime }$ at an optimal level.

**Figure 7:**
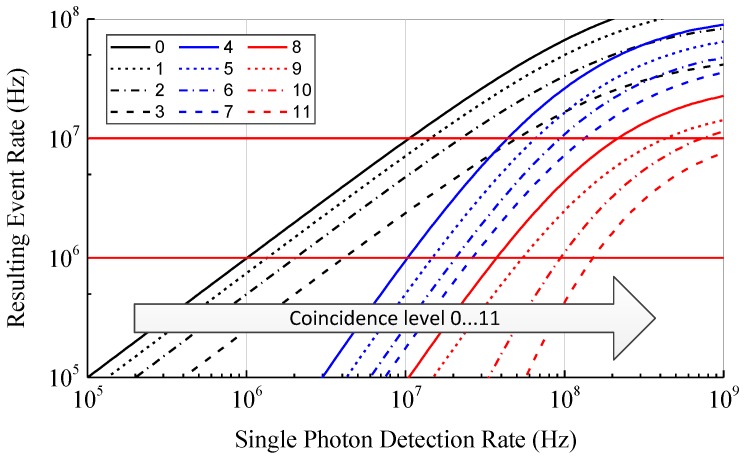
Resulting event rate versus the single photon detection rate according to our theoretical model for the coincidence levels in Table 1. The chosen parameters result in a decreasing event rate between 1 MHz and 10 MHz if passed through in ascending order.

For the implementation of the adaptive coincidence controlling algorithm levels with associated parameters resulting in a decreasing event rate are defined. This is necessary, since increasing the coincidence depth for coarse event rate adjustment and the coincidence time for interpolation results in a non-monotonic behavior of the event rate. The defined coincidence levels and their associated parameters are shown in Table 1. In Figure 7 the resulting event rates of the coincidence levels in Table 1 according to our theoretical model are plotted. The parameters are chosen to get a decreasing resulting event rate in the range from 1 MHz to 10 MHz if the coincidence levels are passed through in ascending order. Due to the different slopes the curves, which depend on the coincidence depth, they intersect at certain points. Therefore, the parameters have to be chosen according to the desired target range of the ambient event rate.

Assuming the distance measurement works well within the defined range of the event rate, the adaptive photon coincidence detection increases the measureable dynamic range in ambient light conditions and target reflectance by more than 40 dB from 20 dB to over 60 dB. Due to the different slopes of the curves, the step size between the coincidence levels depends on the current single photon detection rate. To reduce the step size between the levels, the coincidence parameters need to be varied in finer steps. Since the coincidence depth can only attain integer numbers, this is only possible for the coincidence time. Unfortunately, this requires more complex circuitry.

**Figure 8:**
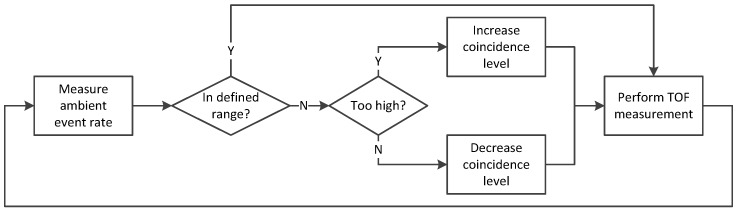
Flow chart of a possible coincidence adjustment algorithm. After the ambient event rate is measured, the coincidence level is increased or decreased if it is outside the defined rate range.

In Figure 8 the flow chart of the current coincidence adjustment algorithm implementation is shown. The first step is measuring the current ambient event rate. In our sensor this is done by using the event counting mode. Since the laser cycle time is 100 µs and the measurement window 1.28 µs, the ambient can be measured in-between consecutive laser pulses without losing frame rate. Next, the measured ambient event rate is compared to the previously defined target range. If the event rate is within the target range, no adjustment of the coincidence parameters is necessary and the distance measurement continues with unchanged settings. If the measured event rate is outside the target range, the parameters are adjusted depending on the actual event rate. In case the event rate is too high, the rate is reduced by increasing the coincidence level. If the rate is too low, the coincidence level is decreased. According to Equation (2) the target range of the event rate has to be chosen according to the desired range of the LiDAR system.

In the presented realization the coincidence levels are varied by one step in each cycle only. A cycle corresponds to one frame, which is 40 ms in the typical setup of the camera. Therefore, in fast-changing environments this kind of coincidence adjustment could be too slow to allow a reliable real-time distance measurement. Assuming a large change in reflectance, the system takes 10 cycles corresponding to 0.4 s to adjust the coincidence settings. To increase the adjustment speed, the step size of the parameter adjustment could be increased in case of high divergence between the actual and target event rates. Another method for the parameter adjustment is to use a look-up table. Here, the best suitable coincidence level is directly chosen based on the measured ambient event rate. For this method the coincidence has to be turned off during the counting mode or the look-up table has to include all possible coincidence levels. This reduces the settling time down to just a single cycle.

## 5. Measurements

**Figure 9:**
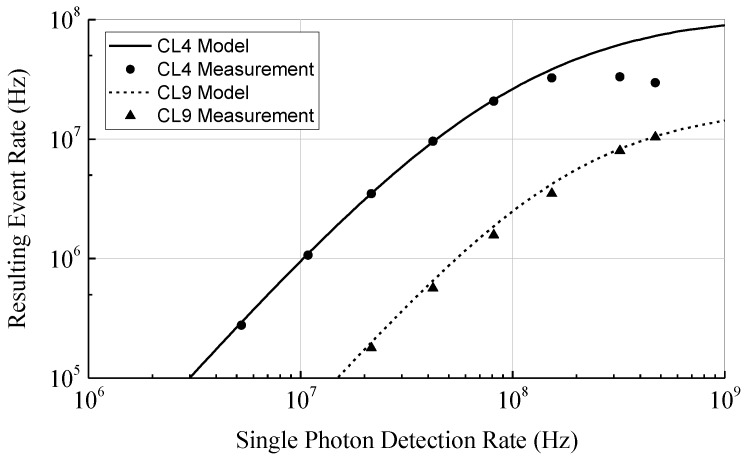
Theoretical coincidence model versus measurement. For a low number of active SPADs and short coincidence time (CL9), the influence of the detection circuits is small and a good agreement is achieved. If more SPADs are used and the coincidence time is close to the dead time (CL4), the measured event rates saturate at higher photon detection rates due to the detection circuit.

The first measurement is to compare the theoretical coincidence model to actual measurements. In Figure 9 the theoretical model and the measurement of the ambient event rates is plotted for the coincidence levels 4 (CL4) and 9 (CL9). For level 9 a good agreement over the whole measurement range can be observed, whereas for level 4 the discrepancy increases at higher photon detection rates. Since the theoretical model does not include the effects of the photon coincidence detection circuit, this behavior is as expected. According to Table 1 in level 9 only two SPADs are used and the coincidence time is set to 4 ns. With these settings an overlap of the pulses generated by the single SPADs is quite unlikely since the dead time is 20 ns and, hence, much longer than the coincidence time. Otherwise in level 4, where four SPADs are activated and the coincidence time of 16 ns is almost as long as the dead time. Therefore, an overlap of the single pulses is much more likely. Since in case of overlapping pulses the output of the coincidence detection circuits stays high, no further events are recorded by the counter. This effect reduces the actual measured event rate at high single photon detection rates as shown in Figure 9. To improve the coincidence controlling algorithm the effect of the coincidence detection circuits has to be included in the theoretical model or the resulting event rates for the different coincidence levels have to be acquired by measurements instead of using the theoretical model.

The further measurements are performed with our 192 × 2 pixel LiDAR sensor mounted in the camera “Owl”. The lens and laser sources are chosen according to the description in the previous section. In the outdoor measurements we use Lambertian targets with different reflectance levels.

**Figure 10:**
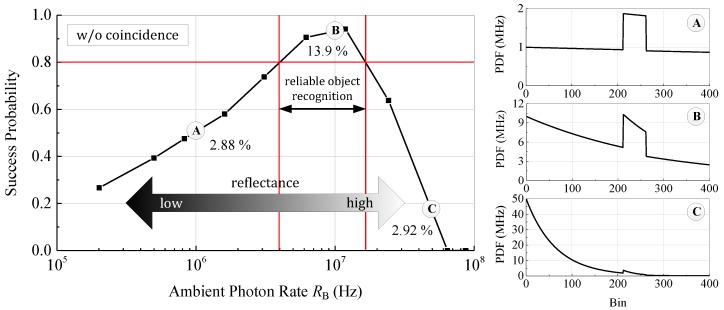
Success probability of the distance measurement at 100 klx ambient sunlight without photon coincidence vs. the background photon rate at constant SBR corresponding to varying target reflectance. Only for a small range in reflectivity a good measurement performance is achieved.

In the next measurement we investigate the effect of varying target reflectance on the system performance. Since the received photon rates of the ambient light and laser signal are proportional to the reflectance, we expect good performance without coincidence only for a limited dynamic range. As a measure of performance, the parameter success probability, defined as the probability to measure the true distance with a maximum deviation of 10%, is calculated from 1000 single distance measurements, whereas each distance is obtained from a histogram filled with 400 timestamps. Obviously, the algorithm to extract the distance from the filled histogram has a major influence on the measurement performance. However, to show the influence of the ambient event rate on the measurement, only the quantitative shape of the measurement curve is of interest and, therefore, an arbitrary algorithm can be applied. We simulate the change in reflectance by adjusting the reception optics aperture. This affects only the absolute intensities of the ambient light and laser while the SBR is kept constant. Figure 10 shows the success probability for an 80% reflectance white Lambertian reflector measured outdoor at 100 klx ambient sunlight without applied photon coincidence and theoretical PDFs for selected values. Since the bin width of 312.5 ps corresponds to a distance of 4.7 cm, the target distance of 10 m corresponds to bin number 212. For high reflectance and photon rates, respectively, the sensor is triggered in almost any measurement by ambient photons. Since the first detected photon stops the time measurement, the sensor is blind at the reception of the laser pulse and a reliable distance measurement is not possible. The probability of measuring the correct time, given by the integral of the PDF over the pulse width, is only 2.92% (PDF C). In the case of low reflectance and photon rates, respectively, also the laser signal is suppressed and only very few signal photons are detected. Similar to the case of high reflectance the probability of a correct time measurement is only 2.88%. Due to the low number of received photons, the noise is high and the laser pulse cannot be located reliably in the histogram (PDF A).

Assuming a minimum required success probability of 80%, only within a dynamic range of around 12 dB of the ambient photon rate $R_{B}$ a reliable distance measurement is possible. In this case the probability of a correct measurement is 13.9% and, thus, much higher than in the other cases (PDF B). In the measurement the target distance was 10 m giving an ideal ambient event rate of $R_{B}=15\;\mathrm{MHz}$ according to Equation (2). The result of the measurement shows a good agreement, even if the maximum can be found at around 12 MHz due the low number of measured points. As mentioned before, typical target scenes in outdoor applications exhibit a high dynamic range in reflectance far above 12 dB and, therefore, the detection rates have to be adjusted pixel-wise to allow a longer range and an accurate measurement of the whole scene.

**Figure 11:**
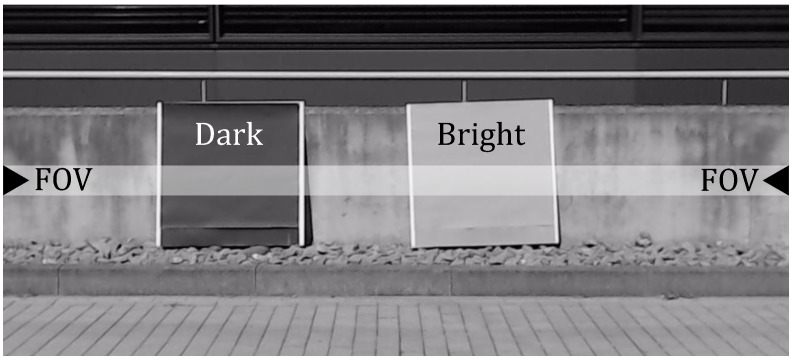
Outdoor target scene measured at 100 klx ambient sunlight including a dark and bright Lambertian target. The distance is measured along the horizontal line in the figure.

Next, a dark (i.e., 8% reflectivity) and a bright (i.e., 60% reflectivity) object, 6.5 m distant, at 100 klx ambient sunlight are measured in a scene to show the capability of the on-chip pixel-wise adaptive coincidence parameter adjustment. Figure 11 shows the measured scene with the dark and bright object. The distance is measured with one line of the sensor along the horizontal line.

**Figure 12:**
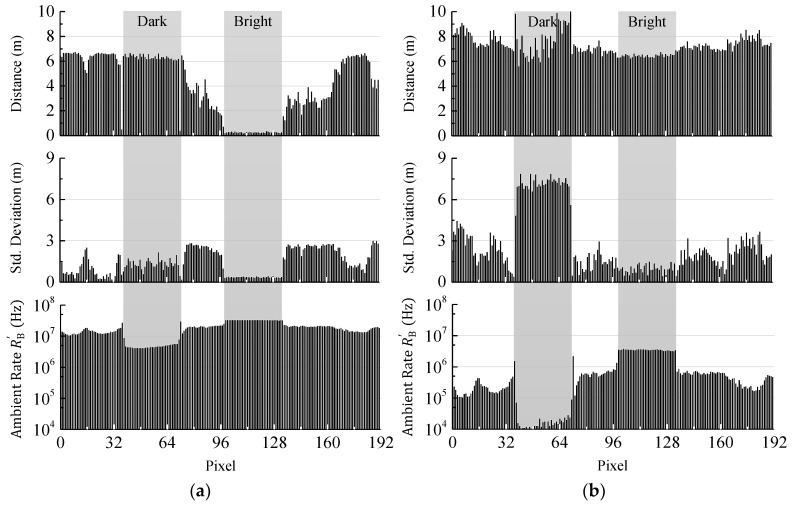
(**a**) Measured mean distance, standard deviation, and ambient event rate for low ambient light rejection. While the dark target can be measured properly, the received event rate of the bright target is too high; (**b**) Same measurement for a high ambient light rejection. In this case the bright target can be measured while the dark one cannot.

In Figure 12a the measured mean distance and standard deviation acquired from 100 single distance measurements along with the ambient event rate $R_{B}^{\prime }$ for each pixel of the sensor line is shown. In this example, the coincidence parameters are identical for the whole pixel line and are set to allow a measurement of the dark object (i.e., coincidence depth of 2 and time of 16 ns). Since the event rate reduction is low, the ambient event detection rate $R_{B}^{\prime }$ at the bright target is too high to allow a reliable measurement (see Figure 10, PDF C). Instead there is a high probability for the time measurement to be stopped by ambient light right at the beginning of the reception window. As a result, a distance close to zero is measured mostly and the standard deviation is quite low. A look at the measured ambient event rates $R_{B}^{\prime }$ shows a rate of around 5 MHz for the dark object and around 33 MHz for the bright one.

In Figure 12b the coincidence parameters are adjusted to allow a measurement of the bright object (i.e., coincidence depth of 4 and time of 16 ns). Here, almost no signal events are received from the dark target making a measurement hardly possible (see Figure 10, PDF A). In this case the determined distance is equally distributed over the whole range and, therefore, the deviation is high. The ambient event rate $R_{B}^{\prime }$ for the bright target is 3.8 MHz while for the dark object only 15 kHz are measured. Additionally, an increase of the event rate dynamic range at higher coincidence depth from a factor of 6.6 (i.e., 16.4 dB) up to a factor of 253 (i.e., 48 dB) is observed. In the low coincidence level case (Figure 12a) the event rate of the bright target is limited by saturation effects, like dead time. As a consequence, the dynamic range of the resulting ambient event rate $R_{B}^{\prime }$ is not increased by applying coincidence. A measurement of both targets shows a difference in reflectivity of 16.5 dB. However, it is shown that achieving high range performance for targets with different reflectance at high ambient illumination is difficult, if identical coincidence parameters are applied.

**Figure 13:**
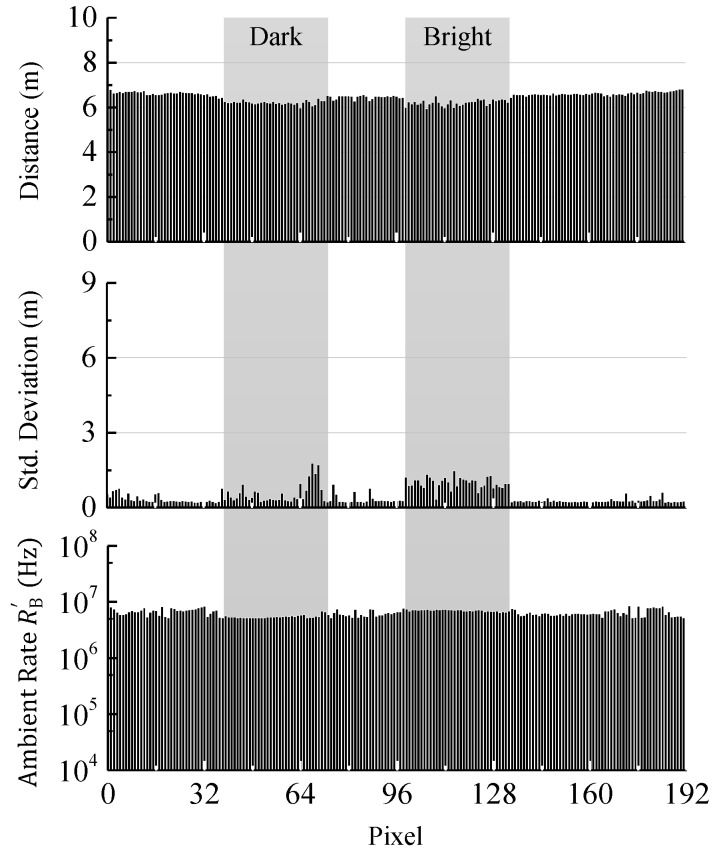
Mean distance, standard deviation, and ambient event rate after applying pixel-wise adaptive coincidence parameter adjustment. This allows a measurement of dark and bright targets in the same scene at once. The ambient event rates are kept at an almost constant level for all pixels.

In Figure 13 the measurement of the scene is shown after applying adaptive photon coincidence detection. Now both targets in the scene can be measured at once. The adaptive photon coincidence algorithm adjusts the coincidence parameters pixel-wise based on the actual ambient event detection rates. The purpose of the parameter adjustment is to get an optimal ambient event rate according to Equation (2), since at this rate the best measurement performance is achieved. In the shown measurement the target window of the ambient event rate lies between 5 MHz and 10 MHz. The results show that the standard deviations for both targets as well as the wall in the background are now as low or even less than in the previous measurements (Figure 12) and the ambient event rates $R_{B}^{\prime }$ are within the defined range. For the bright target, as well as for a part of the dark one the standard deviation is still high compared to the distance. This is due to the algorithm used for extracting the distance from the raw data histogram and will be improved by more sophisticated data processing in the future.

The measurement proves that achieving long range in scenes with high dynamic range in target reflectance and high ambient illumination requires pixel-wise event rate adjustment. The concept of adaptive photon coincidence detection is one promising possibility to fulfill this task and improve the measurement performance. For automotive short and mid-range applications distances up to 50 m are of interest. To extend the range of the shown system several improvements are possible: reducing the width of the optical bandpass filter from 80 nm down to 10 nm reduces the ambient light by a factor of 8. Since the laser pulse intensity scales quadratic with the distance, the range increases by approximately factor 2.5. Another possibility is the usage of laser sources with shorter pulses and higher repetition rates. And also improvements in the histogram processing by the usage of more sophisticated algorithms will extend the system range. Nevertheless, the proposed method of adaptive photon coincidence detection can be implemented in any LiDAR system based on the direct TOF measurement to improve the dynamic range.

## 6. Conclusions

To enable a reliable distance measurement with CMOS SPAD-based LiDAR systems at strong ambient illumination, background light suppression is essential. Without the application of any ambient light rejection method, an improvement of the SPAD sensitivity is not generally beneficial in the first-photon direct TOF measurement, because higher PDE can imply a higher probability of unwanted background photon detections and, hence, false measurements. The detection of photon coincidences reduces the probability for background generated events and improves the ability to separate between ambient light and laser signal. This allows for a more reliable measurement. Since the best measurement performance is achieved only for a specific and distance dependent ambient event rate, the adjustment of the event rate by varying the coincidence parameters enables the sensor to capture scenes with a high dynamic range in object reflectance. To be applicable in real-world traffic situations, the parameter adjustment needs to be performed in real time. This can be achieved by using dynamic step size of the parameter adjustment or a look-up table approach. Since applying photon coincidence detection reduces the resulting event rates and shows a dead-time induced saturation, a higher SPAD sensitivity and lower dead-time can further improve the advantages of photon coincidence detection.

## Figures and Tables

**Table 1:** Coincidence parameters of the chosen coincidence level for decreasing event rate.

Coincidence Level	0	1	2	3	4	5	6	7	8	9	10	11
Coincidence Depth	1	1	1	1	2	2	2	2	2	2	3	4
Coincidence Time (ns)	-	-	-	-	16	16	8	16	8	4	4	8
Number of SPADs	4	3	2	1	4	3	3	2	2	2	3	4
